# Potential of Carbon Nanotube Chemiresistor Array in Detecting Gas-Phase Mixtures of Toxic Chemical Compounds

**DOI:** 10.3390/nano13152199

**Published:** 2023-07-28

**Authors:** Seongwoo Lee, Sanghwan Park, Seongyeop Lim, Cheongha Lee, Chang Young Lee

**Affiliations:** 1 Department of Materials Science and Engineering, Ulsan National Institute of Science and Technology (UNIST), Ulsan 44919, Republic of Korea; lsw911218@unist.ac.kr; 2 School of Energy and Chemical Engineering, Ulsan National Institute of Science and Technology (UNIST), Ulsan 44919, Republic of Korea; sanghwanpark@unist.ac.kr (S.P.); lsy981202@unist.ac.kr (S.L.); cheongha@unist.ac.kr (C.L.); 3 Graduate School of Carbon Neutrality, Ulsan National Institute of Science and Technology (UNIST), Ulsan 44919, Republic of Korea

**Keywords:** gas sensor array, single-walled carbon nanotubes, toxic industrial chemicals, adsorption

## Abstract

Toxic industrial chemicals (TICs), when accidentally released into the workplace or environment, often form a gaseous mixture that complicates detection and mitigation measures. However, most of the existing gas sensors are unsuitable for detecting such mixtures. In this study, we demonstrated the detection and identification of gaseous mixtures of TICs using a chemiresistor array of single-walled carbon nanotubes (SWCNTs). The array consists of three SWCNT chemiresistors coated with different molecular/ionic species, achieving a limit of detection (LOD) of 2.2 ppb for ammonia (NH_3_), 820 ppb for sulfur dioxide (SO_2_), and 2.4 ppm for ethylene oxide (EtO). By fitting the concentration-dependent sensor responses to an adsorption isotherm, we extracted parameters that characterize each analyte-coating combination, including the proportionality and equilibrium constants for adsorption. Principal component analysis confirmed that the sensor array detected and identified mixtures of two TIC gases: NH_3_/SO_2_, NH_3_/EtO, and SO_2_/EtO. Exposing the sensor array to three TIC mixtures with various EtO/SO_2_ ratios at a fixed NH_3_ concentration showed an excellent correlation between the sensor response and the mixture composition. Additionally, we proposed concentration ranges within which the sensor array can effectively detect the gaseous mixtures. Being highly sensitive and capable of analyzing both individual and mixed TICs, our gas sensor array has great potential for monitoring the safety and environmental effects of industrial chemical processes.

## 1. Introduction

The detection and identification of toxic industrial chemicals (TICs) are essential for workplace safety, public health, and environmental monitoring [1,2]. Several gas sensors have been developed using various nanomaterials such as carbon nanotubes (CNTs) [3,4], graphene [5,6], nanowire [7], semiconducting materials [8,9,10] and metal–organic frameworks [11] because of their high surface-to-volume ratios and sensitivity to chemical environments. CNTs, in particular, have a one-dimensional electronic structure, where all atoms reside only on the surface and are extremely sensitive to molecular adsorption [12,13]. However, most previous studies have focused on improving the sensitivity and selectivity toward a single gaseous analyte rather than the analysis of multi-analyte mixtures.

To overcome the limitations of detecting and identifying complex gas mixtures, advanced technologies have been developed, such as gas sensor arrays based on nanomaterials [14,15,16,17,18,19,20,21,22,23]. For example, Guerin et al. demonstrated a CNT chemiresistor array with an electrode–CNT interface-derived sensitivity difference for H_2_, NH_3_, toluene, and ethanol using various metal electrodes (Pt, Pd, and Au) [24]. Yi et al. reported a gas sensor array using a nanowire-like network film of ZnO, Co_3_O_4_, IN_2_O_3_, and SnO_2_ that enables the selective detection and identification of C_7_H_8_, NH_3_, HCHO, and CH_3_COCH_3_ [25]. Although previous studies have demonstrated excellent selectivity for single gaseous analytes, the sensing performance for mixture analysis has not been validated yet. Chu et al. recently reported reliable identification of mixtures using sensor arrays [26]. However, complex numerical analysis and the assistance of neural networks are necessary [26,27,28,29,30,31].

In this work, the detection and identification of gaseous mixtures of toxic chemicals were enabled using a single-walled carbon nanotube (SWCNTs) chemiresistor array. We chose ammonia (NH_3_), sulfur dioxide (SO_2_), and ethylene oxide (EtO) as target analyte gases. The selection of these gases was based on the fact that each individual analyte is a hazardous gas, and their mixture poses a significant threat to the environment and safety. For example, in a moisturized environment, the reaction between NH_3_ and SO_2_ can lead to the formation of acid rain [32,33,34]. Furthermore, the reaction between NH_3_ and EtO has the potential to result in an explosion [35,36,37]. Different adsorption/desorption properties of gas molecules on SWCNTs were obtained using polymeric and ionic chemical coatings, thus allowing identification of the three TICs—NH_3_, SO_2_, and EtO—alone or as mixtures. In addition, by analyzing the concentration-dependent response curves, we estimated parameters that govern the analyte adsorption, such as equilibrium constant and limits of detection (LOD) of each analyte. Our sensor array has the potential to be applied to a wide range of multi-analyte sensing systems for environmental monitoring and alarm systems to prevent accidents and minimize the potential harm caused by exposure to hazardous chemicals.

## 2. Materials and Methods

### 2.1. Materials

All chemicals and reagents were commercially available, and we utilized these without additional purification. 1-ethyl-3-methylimidazolium bis(trifluoro-methylsulfonyl)imide (EMIM), polypyrrole (Ppy), dimethyl sulfoxide (DMSO), and phosphate buffered saline (PBS) were purchased from Sigma-Aldrich. The gas cylinders for NH_3_ (10 ppm), SO_2_ (76.3 ppm), and EtO (666 ppm) were purchased from RIGAS (Daejeon, Korea). Graphing and PCA analysis were achieved by using Origin software (OriginPro 2020, OriginLab Corp., Northampton, MA, USA)

### 2.2. Sensor Fabrication

Pristine SWCNTs (AP-SWNT, Carbon Solutions, Inc., Riverside, CA, USA) were dispersed in a 1 wt% aqueous solution of sodium dodecyl sulfate (Sigma-Aldrich, St. Louis, MO, USA) by homogenization (6600 rpm, 1 h), followed by a bath sonication for 1 h. The dispersion was then centrifuged (14,000 rpm for 1 h) to remove large aggregates, and a homogeneous dispersion was obtained by collecting the supernatant. The concentration of the SWCNT dispersion was estimated to be 16.5 mg/L by UV-VIS-NIR absorbance at 632 nm (Cary 5000, Agilent Technologies, Santa Clara, CA, USA) and an extinction coefficient of ε_632_ = 0.036 (mg/L)^−1^ cm^−1^ (Figure S1) [38]. The 25 μL of SWCNT dispersion was vacuum filtered through a polycarbonate membrane with 0.2 μm pores (GTTP02500, MERCK, St. Louis, MO, USA) and transferred onto a silicon substrate with 300 nm thermal oxides (DASOM RMS, Anyang, Korea) as reported previously [39].

The sensor array was fabricated by patterning electrodes on the SWCNT networks. First, extra nanotubes that were not required were removed by conventional photolithography and oxygen plasma etching (100 W, 30 sccm O_2_, 30 s) to reduce the signal interference between the sensors. The density of the SWCNT network was estimated by the amount of SWCNTs (16.5 mg/L × 25 μL) and the area of the SWCNT film (1.95 mm^2^). Then, interdigitated electrodes (2 nm Cr, 75 nm Au) were patterned on the SWCNT networks via the lift-off process. Finally, a structure of 70 μm-thick SU8-2050 was patterned around each sensor, which served as a well for drop-drying coating materials.

**Figure 1:**
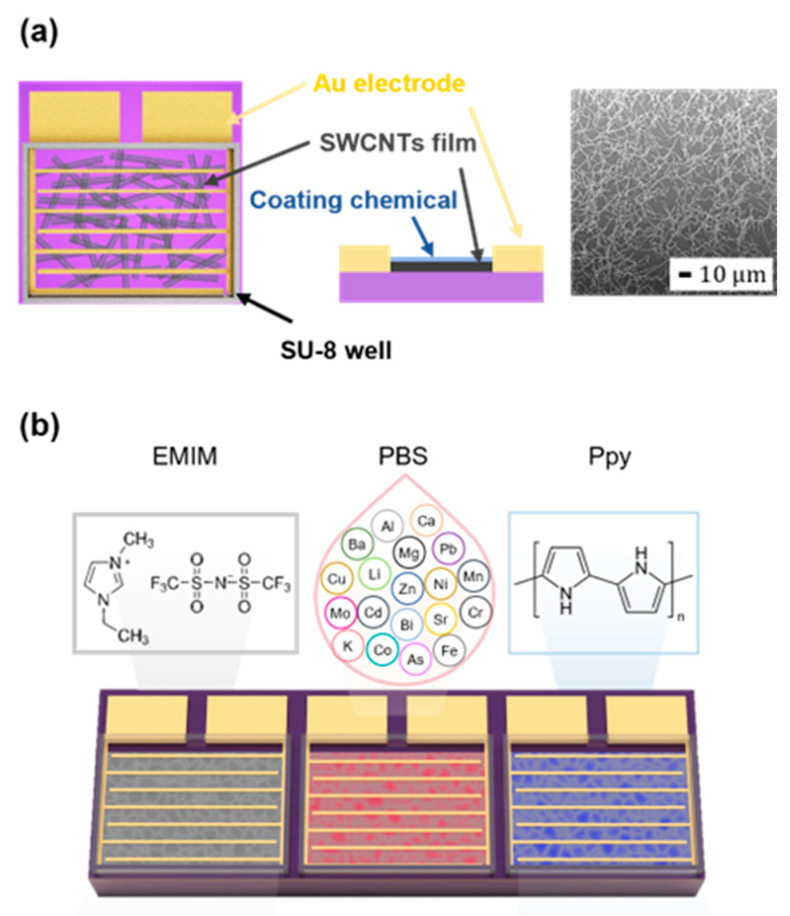
SWCNT sensor array fabrication. (**a**) Design of the SWCNT chemiresistor and SEM images for transferred SWCNT film on SiO_2_ substrate. (**b**) Chemiresistor array coated with EMIM, PBS, and Ppy, respectively.

Figure 1a illustrates the design of the SWCNT chemiresistor. The SEM image depicted in Figure 1a shows the SWCNT network on the SiO_2_ substrate at the density of 0.43 ng/mm^2^. The sensor array consisted of three SWCNT chemiresistors coated with EMIM, PBS, and Ppy (Figure 1b). For the selection of coating materials, we conducted screening experiments using commercially available chemicals that were reported to exhibit high sensitivity and selectivity to the target analyte gases. Based on the sensor response to NH_3_, SO_2_, and EtO (Figure S2), we chose EMIM, PBS, and Ppy as the coating materials. The dimensions of the sensor array used in this study were 2 mm × 6 mm. The sensor coating was performed as follows: For the EMIM and PBS coatings, 0.1 μL of 10 mg/mL EMIM in DMSO solution and 0.1 μL of PBS solution, respectively, was dropped on the sensor and dried in a vacuum desiccator for 4 h. For Ppy coating, 0.1 μL of Ppy was dropped on the sensor and thereafter washed with deionized water and dried with N_2_. The coated-array sensor was placed in a chip carrier, and thereafter, electrically connected to the Au-pad using a wire bonder.

### 2.3. Experimental Setup

**Figure 2:**
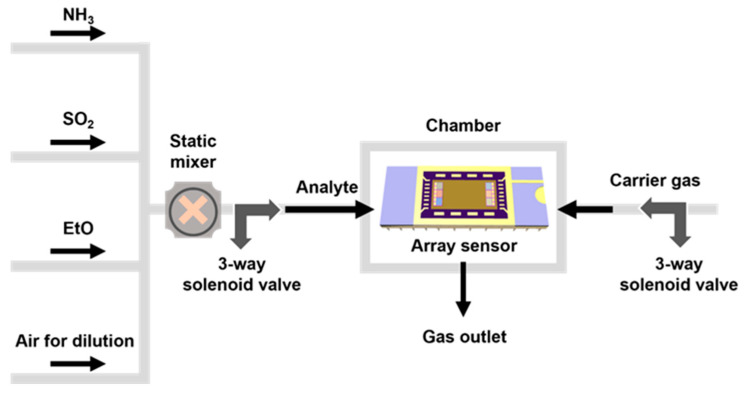
Experimental setup for measuring the performance of SWCNT chemiresistor array.

Figure 2 shows the experimental setup for the gas sensing performance measurement. The analyte gases (NH_3_, SO_2_, EtO) and air cylinders were connected to a mass flow controller (MFC, Kofloc Corp., Kyoto, Japan) to control the on/off status and flow rate of the gas. The total flow rate was fixed at 500 sccm. To control the analyte gas concentration, the carrier gas was balanced using the MFC. A static mixer was used for the uniform mixing of the analyte gases. The resistance of the sensor was measured using a system switch/multimeter (3706A, Keithley, Cleveland, OH, USA). Prior to gas sensing, the sensor array was stabilized in the chamber using carrier gas for 60 s, and the change in resistance was measured by exposing it to the analyte gas for 20 s.

## 3. Results and Discussion

### 3.1. Gas Sensing Performance

**Figure 3:**
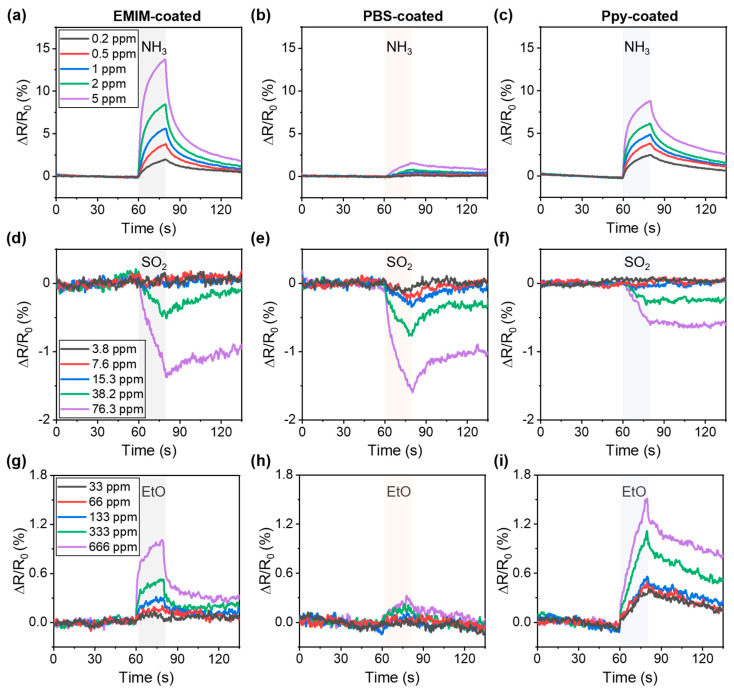
Gas sensing performance of SWCNT chemiresistor arrays. Sensor responses to 0.2–5 ppm NH_3_ exposure of (**a**) EMIM-coated sensor, (**b**) PBS-coated sensor, and (**c**) Ppy-coated sensor. Sensor responses to 3.8–76.3 ppm SO_2_ of (**d**) EMIM-coated sensor, (**e**) PBS-coated sensor, (**f**) Ppy-coated sensor. Sensor responses to 33–666 ppm EtO of (**g**) EMIM-coated sensor, (**h**) PBS-coated sensor, (**i**) Ppy-coated sensor.

Figure 3 shows the gas sensing performance of the SWCNT chemiresistor array. The sensor response (S) is defined as $S=\left(\Delta R/R_{0}\right)/R_{0}\times 100$, where $R_{0}$ and R represent the resistances of the SWCNT chemiresistor before and after exposure to the analytes, respectively. The measurements of sensor response with increasing gas concentration were performed in one single device. For the purpose of minimizing exposure to hazardous gases and providing rapid alarms, the sensor array was exposed to the analyte gases for only 20 s. Although the resistances of the baseline were not fully recovered, and the deviation was less than 1% (Figure S3), small differences in baseline still existed because of the baseline drift of the SWCNT sensors [40,41].

First, we investigated the sensor responses when exposed to a concentration range of 0.2–5 ppm NH_3_, which is lower than the concentration range of NH_3_ immediately dangerous to life or health (IDLH) (300 ppm) [42]. The resistance of the EMIM-coated sensor rapidly increased when exposed to NH_3_, owing to the electron-donating characteristics of NH_3_ on the p-doped SWCNT; the resistance decreased when the exposure to NH_3_ ceased (Figure 3a) [43,44,45]. The sensor responses increased from 2.18% to 13.1% as the concentration of NH_3_ increased from 0.2 ppm to 5 ppm. For the PBS- and Ppy-coated sensors, the increase in resistance was 0.13–1.42% (Figure 3b) and 2.27–8.24% (Figure 3c), respectively, as the concentration of NH_3_ increased from 0.2 ppm to 5 ppm.

In contrast to the sensor responses to NH_3_, the sensors exhibited a decreased resistance to SO_2_ exposure, owing to the oxidizing property of SO_2_ on SWCNTs [46]. Figure 3d–f depict the sensor response of EMIM, PBS, and Ppy-coated sensors, respectively, to 3.8–76.3 ppm of SO_2_ gas. Note that the IDLH of SO_2_ was 100 ppm [42]. In the case of EMIM- and Ppy-coated sensors, sensor responses were observed at 38.2 and 76.3 ppm, respectively, whereas no responses were observed at lower concentrations of SO_2_ (Figure 3d,f). For the PBS-coated sensor, the change in resistance decreased from −0.11% to −1.53% as the concentration of SO_2_ increased from 3.8 ppm to 76.3 ppm (Figure 3e).

Because the IDLH of EtO is 500 ppm, the sensor responses were obtained in the concentration range of 33–666 ppm for the EtO sensing performance investigation. Since EtO is a reducing gas on SWCNTs [47,48], the EMIM-coated sensor exhibited an increased resistance from 0.09% to 1.0% under EtO exposure (Figure 3g). For the PBS-coated sensor, sensor responses from 0.013% to 0.26% were obtained in the concentration range of 66–666 ppm, whereas no response was observed at a concentration of 33 ppm (Figure 3h). The Ppy-coated sensor exhibited an increase in response from 0.37% to 1.40% over the full concentration range (Figure 3i). A comparison of the characteristics of chemiresistors based on nanomaterials is presented in Table 1. Our sensor array allows the detection of target analytes at concentrations under IDLH even after storage for 6 months in an ambient environment (Figure S4).

**Figure 4:**
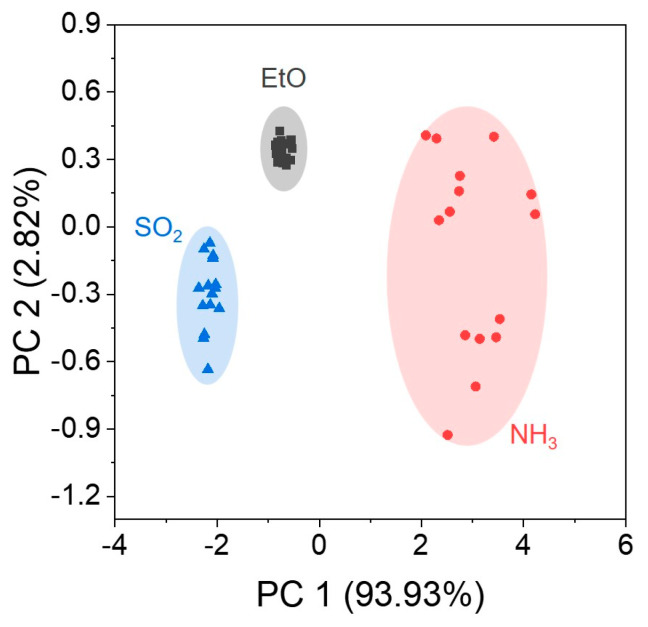
PCA plot of the sensor array response patterns toward single gaseous analytes (*n* ≥ 15).

Based on the results depicted in Figure 3, we demonstrated that the sensor arrays composed of EMIM, PBS, and Ppy-coated SWCNT networks successfully detected NH_3_, SO_2_, and EtO at a lower concentration when compared to the IDLH range, and different sensitivities were obtained depending on the coating chemicals. Thereafter, we performed a principal component analysis (PCA) based on the response patterns of the sensor array under exposure to single species of gaseous analytes. Figure 4 depicts the PCA plot, clearly demonstrating that the sensor array’s response to NH_3_, SO_2_, and EtO was distinctly separated without any overlap. Although each sensor in the sensor array showed imperfect specificity toward single target gaseous analytes, the PCA results suggested that our sensor array allows identification of the analyte molecules.

### 3.2. Adsorption Parameters of SWCNT Sensor

**Figure 5:**
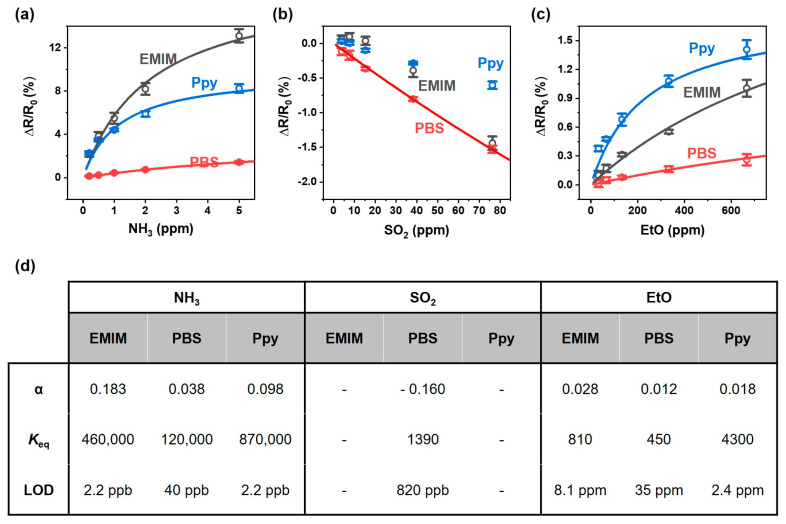
Calibration curve of the response of SWCNT chemiresistor arrays to (**a**) NH_3_, (**b**) SO_2_, (**c**) EtO. (**d**) The parameters of analyte adsorption on the SWCNTs extracted from calibration curve.

Figure 5 depicts the calibration curve of the response of SWCNT chemiresistor arrays to NH_3_, SO_2_, and EtO. In the case of the response to NH_3_, the sensor responses increased almost linearly as the concentration of NH_3_ increased for the low concentration range (<1 ppm), whereas the increase in the sensor response tapered off and was saturated for the high concentration range (>1 ppm) (Figure 5a). For the responses to SO_2_, the resistance decreased linearly with respect to the SO_2_ concentration range of 3.8–76.3 ppm (Figure 5b). Regarding the responses to EtO, the Ppy-coated sensor exhibited the largest increase in sensor resistance, followed by the EMIM- and PBS-coated sensors. Similarly to the sensor response to NH_3_ exposure, the resistance of the sensors demonstrated a linear increase for concentrations lower than 200 ppm, whereas saturated responses were obtained for concentrations greater than 200 ppm (Figure 5c).

Because the sensor response ($\Delta R/R_{0}$) originates from the charge transfer between SWCNTs and adsorbed analytes, the response can be described by the following Langmuir adsorption isotherm [57]:

$$\frac{\Delta R}{R_{0}}=\alpha \frac{K_{eq}\left[A\right]}{1+K_{eq}\left[A\right]}$$

where α is the proportionality factor associated with maximum resistance change at high analyte concentration, $K_{eq}$ is the equilibrium constant for adsorption, $\left[A\right]$ is the analyte concentration. These parameters can be extracted by fitting the concentration-dependent sensor responses to the isotherm, as indicated by the thick solid lines in Figure 5a–c. The extracted parameters are tabulated in Figure 5d. For sensing NH_3_, $K_{eq}$ values of 460,000, 120,000, and 870,000 were obtained for the EMIM-, PBS-, and Ppy-coated SWCNT sensors, respectively. In the case of SO_2_ sensing, a $K_{eq}$ value of 1390 was obtained only from the PBS-coated sensor because the responses of the EMIM- and Ppy-coated sensors could not be obtained for the low concentration range of the analyte (<15.3 ppm). In the case of EtO sensing, $K_{eq}$ values of 810, 450, and 4300 were obtained from the EMIM, PBS, and Ppy- coated SWCNT sensors, respectively. The $K_{eq}$ value was higher for the adsorption of NH_3_ on SWCNTs when compared to values for the adsorption of SO_2_ and EtO. In addition, the Ppy-coated sensor exhibited a significantly increased $K_{eq}$ value for the adsorption of EtO when compared to the EMIM- and PBS-coated sensors. It should be noted that the responses shown in Figure 3 did not reach complete equilibrium, meaning that the α and $K_{eq}$ values reported in in this work are underestimated.

The limit of detection (LOD) can be estimated by extrapolating the response curves in Figure 5a–c down to three times the noise level, which we define as the standard deviation of Δ*R*/*R*_0_ prior to exposure to the analytes. The LOD values were obtained as follows: 2.2 ppb for NH_3_ from the EMIM- and Ppy-coated sensors, 820 ppb for SO_2_ from the PBS-coated sensor, and 2.4 ppm for EtO from the Ppy-coated sensor.

### 3.3. Sensor Response to Mixtures of Gas Molecules

We investigated whether the chemiresistor array response could be used to analyze a mixture of gaseous chemicals. To understand the response of the sensor to the analyte mixture, which varied depending on the composition of the mixture, the responses to a 250:250 sccm of two species were investigated. Note that the concentration ratio was 5:38.2 ppm for NH_3_:SO_2_, 5:333 ppm for NH_3_:EtO, and 38.2:333 ppm for SO_2_:EtO mixture.

**Figure 6:**
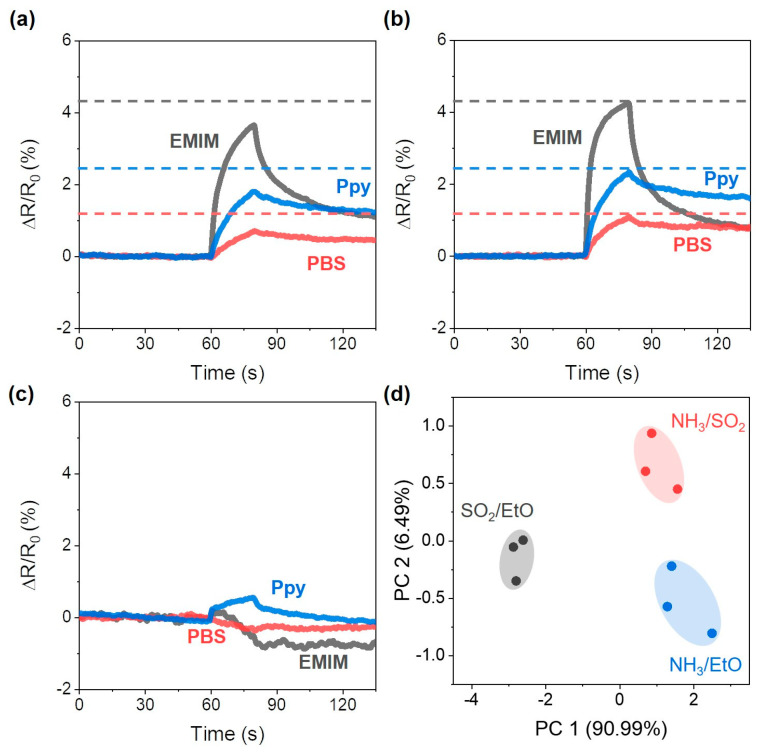
Sensor array response to (**a**) NH_3_/SO_2_ mixture, (**b**) NH_3_/EtO mixture, (**c**) SO_2_/EtO mixture. (**d**) PCA plot of the sensor array response patterns when exposed to the analyte mixture.

The sensor array responses to the NH_3_/SO_2_ mixture were 3.66%, 0.70%, and 1.80% for the sensors coated with EMIM, PBS, and Ppy, respectively (Figure 6a). In the case of sensor array responses to the mixture of NH_3_/EtO, the responses were 4.26%, 1.11%, and 2.33% for the sensors coated with EMIM, PBS, and Ppy, respectively (Figure 6b). Because of the significantly larger $K_{eq}$ value of NH_3_ adsorption on the SWCNTs, both sensor array responses seemed to be similar to the sensor responses to NH_3_ alone (Figure 3a–c). However, all of the sensor responses to the NH_3_/EtO mixture were larger than those to the NH_3_/SO_2_ mixture. Because SO_2_ exposure resulted in decreased resistance of the sensors (Figure 3d–f), the resistance increased with respect to the EtO exposure (Figure 3g–i); the difference in sensor response to the mixture was due to a difference in mixture content. As depicted in Figure 6c, in the case of the sensor response to the SO_2_/EtO mixture, the resistance of the EMIM-coated sensor showed a decreased response (−0.72%) after the gas exposure, and the PBS-coated sensor showed a slightly decreased resistance (−0.38%), while the Ppy-coated sensor showed a slightly increased resistance (0.56%). In contrast to the mixture containing NH_3_, which had a high $K_{eq}$ (>460,000), the competitive adsorption of the analyte was expected to depend on the value of $K_{eq}$ for SO_2_ and EtO adsorption on the SWCNTs array. For example, the response of the PBS-coated sensor showed a decreased resistance to the SO_2_/EtO mixture (red), owing to the larger $K_{eq}$ value of SO_2_ (1390) when compared to that of EtO (450). On the other hand, the $K_{eq}$ value of SO_2_ adsorption could not be obtained for the EMIM- and Ppy-coated sensors; however, we could estimate the $K_{eq}$ value of SO_2_ adsorption. For example, we can assume that the $K_{eq}$ value of SO_2_ adsorption on EMIM-coated SWCNTs would be larger than 810, which was the $K_{eq}$ value of EtO adsorption, because the resistance of the EMIM-coated sensor decreased with exposure to the SO_2_/EtO mixture (Figure 6c), even when the resistance of the EMIM-coated sensor increased with exposure to pure EtO (Figure 3g). On the other hand, the $K_{eq}$ value of SO_2_ adsorption on the Ppy-coated sensor was expected to be smaller than 4300, which was the value of the $K_{eq}$ of EtO adsorption. As indicated by the blue curve in Figure 6c, the resistance increased after exposure to the SO_2_/EtO mixture; thus, the EtO adsorption was more dominant when compared to SO_2_ adsorption on the Ppy-coated SWCNTs. To discriminate the analyte mixtures, we performed the PCA analysis based on the response patterns of the sensor array under exposure to the analyte mixture. The PCA plot depicted in Figure 6d indicates that the responses of the sensor array to mixtures of NH_3_/SO_2_, NH_3_/EtO, and SO_2_/EtO were clearly differentiated into three groups without overlap.

**Figure 7:**
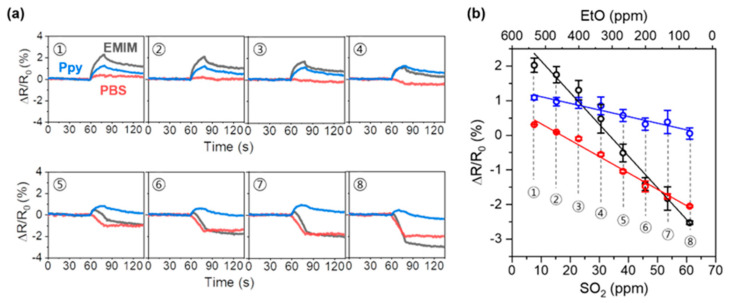
Detection of three-analyte mixtures. (**a**) Sensor array response to the mixtures of NH_3_, SO_2_, and EtO with content variations, and (**b**) linear fitting of sensor array response to the concentration of gas mixture.

To demonstrate the application of our sensor array system for the analysis of complex mixtures, we examined the responses of the sensor array to a three-species mixture of NH_3_, SO_2_, and EtO (Figure 7). Because the $K_{eq}$ value of NH_3_ adsorption is significantly larger than the $K_{eq}$ values of SO_2_ and EtO adsorption, dominant NH_3_-derived sensor responses were observed for the mixtures that contained high NH_3_ content. Figure 7a shows the response of the sensor array to mixtures of NH_3_, SO_2_, and EtO with varying contents. It should be noted that the concentration of NH_3_, [NH_3_], was fixed to 1 ppm, and the concentration of of SO_2_, [SO_2_], increased from 7.63 to 61.04 ppm, whereas the concentration of EtO, [EtO], decreased from 532.8 to 66.6 ppm. In the case of the EMIM-coated sensor (black), a large increase in resistance (2.31%) was observed at [SO_2_] = 7.63 ppm, and a significant decrease was observed in the increase in resistance when [SO_2_] increased, until it reached 30.52 ppm (1.06%). The EMIM-coated sensor exhibited a negative response (−0.14%) at [SO_2_] = 38.2 ppm, and the decrease in resistance increased as [SO_2_] increased to 61.04 ppm (−2.48%). For the PBS-coated sensor (red), which showed a small increase in resistance upon exposure to NH_3_ and EtO (Figure 3b,h), a slightly increased resistance was observed (0.30%) when [SO_2_] = 7.63 ppm. Then, the resistance of the sensor continuously decreased to −2.07% as the [SO_2_] increased to 61.2 ppm. In the case of the Ppy-coated sensor (blue), which had sensitive responses to NH_3_ and EtO exposure (Figure 3c,i), the resistance increased to 1.18% at [SO_2_] = 7.63 ppm. Then, with an increase in [SO_2_] from 7.63 to 61.04 ppm, the response decreased to 0.28%. We further investigated the responses of the sensor array when the NH_3_ concentration was higher than 1 ppm (Figure S5). As the PBS-coated sensor is sensitive to SO_2_ gas, the SO_2_ and EtO in the mixture seemed to be detected. However, the detection capabilities of our sensor array seemed to be limited due to the dominant sensor responses to NH_3_ when the concentration of NH_3_ exceeds 5 ppm. Nevertheless, the expected detectable concentration range for SO_2_ was 38.2–53.41 ppm, and for EtO, it was 333–466.2 ppm. It is worth noting that these concentration ranges for both SO_2_ and EtO are still lower than their IDLH concentrations. The sensor array responses were plotted as a function of the gas mixture composition (Figure 7b). As the concentration of SO_2_ increased, the response of the sensor array decreased linearly. From the linear plot of the sensor array responses, we obtained the relationships between sensor response and concentration of SO_2_ in our experimental system with high R^2^ values (R^2^ = 0.982, 0.986, and 0.941 for the EMIM, PBS, and Ppy-coated sensors, respectively).

$$S_{EMIM}\left(\%\right)=-9.167\times 10^{4}\;\left[SO_{2}\right]+3.07$$

$$S_{PBS}\left(\%\right)=-4.736\times 10^{4}\;\left[SO_{2}\right]+8.07$$

$$S_{Ppy}\left(\%\right)=-1.914\times 10^{4}\;\left[SO_{2}\right]+1.31$$

The relationship can also be described in terms of EtO concentration (R^2^ = 0.982, 0.986, and 0.941 for the EMIM, PBS, and Ppy-coated sensors, respectively).

$$S_{EMIM}\left(\%\right)=1.529\times 10^{4}\;\left[EtO\right]-3.245$$

$$S_{PBS}\left(\%\right)=0.543\times 10^{4}\;\left[EtO\right]-2.453$$

$$S_{Ppy}\left(\%\right)=0.219\times 10^{4}\;\left[EtO\right]-0.001$$

As the sensor array response showed a linear correlation with the mixture composition, we expect that our sensor array system has potential for the component analysis of the TIC mixtures.

## 4. Conclusions

In summary, we developed a highly sensitive SWCNT-based chemiresistor array for TIC gas sensing. The coating of EMIM, PBS, and Ppy on the SWCNT networks successfully tuned the sensitivity to NH_3_, SO_2_, and EtO by changing the characteristics of the analyte molecules’ adsorption onto the SWCNT surfaces. Our sensor array not only provided an extremely low limit of detection for a single gaseous analyte, but also allowed the detection and identification of a mixture of gaseous TICs. In addition, the linear correlation of the sensor array responses to component variations in multi-analyte mixtures suggests high potential for the real-time monitoring of TIC gases with qualitative and quantitative component analysis.

## Figures and Tables

**Table 1:** A comparison of the characteristics of chemiresistor based on nanomaterials.

Gas	Materials	Concentration	Response	Year	Ref.
NH_3_	CNT coated with EMIM	0.2 ppm	2.18%	2023	This work
	sc-SWCNTs, m-SWCNTs	5 ppm	18%	2022	[49]
	p-SWCNTs, f-SWCNTs	8 ppm	5.8%	2020	[50]
	SWCNTs -OH/PANI	100 ppm	14.91%	2019	[51]
	COOH-functionalized SWCNTs	300 ppm	30%	2018	[52]
SO_2_	CNT coated with PBS	3.8 ppm	0.11%	2023	This work
	SnO_2_/CNT	1 ppm	2.3%	2022	[53]
	MWCNT/MoS_2_	0.5 ppm	0.22%	2021	[54]
	Ni-MOF/ –OH-SWNTs	0.5 ppm	~1.8%	2021	[55]
EtO	CNT coated with Ppy	33 ppm	0.37%	2023	This work
	0.5 wt% Sm_2_O_3_-doped SnO_2_ nanoparticles	30 ppm	61.9 at 350 °C	2018	[56]

## Data Availability

Data presented in this study are available on request from the corresponding author.

## Supplementary Materials

The following are available online at https://www.mdpi.com/article/10.3390/nano13152199/s1, Figure S1 Coating material screening result compared to pristine SWCNTs sensor. Figure S2 Coating material screening result compared to pristine SWCNTs sensor. Figure S3 Baseline of EMIM-coated sensors after NH3 exposures. Figure S4. Stability of the sensor array. Sensor responses to NH3, SO2, and EtO were obtained after 6 months storage. Figure S5 Sensor array responses toward three species gas mixtures.
