# Supplementary material for: Potential of Carbon Nanotube Chemiresistor Array in Detecting Gas-Phase Mixtures of Toxic Chemical Compounds
Source: Nanomaterials (Basel). 2023 Jul 28;13(15):2199. doi: 10.3390/nano13152199 (PMC10421483; doi:10.3390/nano13152199)
Supplement: Supplementary file 1 [file nanomaterials-13-02199-s001.zip › nanomaterials-2477620-supplementary.pdf]

Supporting Information

Potential of Carbon Nanotube Chemiresistor Array in Detecting Gas-Phase Mixtures of Toxic Chemical Compounds

Seongwoo Lee [1], Sanghwan Park [2], Seongyeop Lim [2], Cheongha Lee [2] and Chang Young Lee [2,3,*]

[1] Department of Materials Science and Engineering, Ulsan National Institute of Science and Technology (UNIST), Ulsan 44919, Republic of Korea; lsw911218@unist.ac.kr

[2] School of Energy and Chemical Engineering, Ulsan National Institute of Science and Technology (UNIST), Ulsan 44919, Republic of Korea; sanghwanpark@unist.ac.kr (S.P.); lsy981202@unist.ac.kr (S.L.); cheongha@unist.ac.kr (C.L.)

[3] Graduate School of Carbon Neutrality, Ulsan National Institute of Science and Technology (UNIST), Ulsan 44919, Republic of Korea

* Correspondence: cylee@unist.ac.kr

Abstract: Toxic industrial chemicals (TICs), when accidentally released into the workplace or environment, often form a gaseous mixture that complicates detection and mitigation measures. However, most of the existing gas sensors are unsuitable for detecting such mixtures. In this study, we demonstrated the detection and identification of gaseous mixture of TICs using a chemiresistor array of single-walled carbon nanotubes (SWCNTs). The array consists of three SWCNTs chemiresistors coated with different molecular/ionic species, achieving limit of detection (LOD) of 2.2 ppb for ammonia (NH_3), 820 ppb for sulfur dioxide (SO_2), and 2.4 ppm for ethylene oxide (EtO). By fitting the concentration-dependent sensor responses to an adsorption isotherm, we extracted parameters that characterize each analyte-coating combination, including the proportionality and equilibrium constants for adsorption. Principal component analysis confirmed that the sensor array detected and identified a mixture of two TIC gases: NH_3/SO_2, NH_3/EtO, and SO_2/EtO. Exposing the sensor array to three TIC mixtures with various EtO/SO_2 ratios at a fixed NH_3 concentration showed an excellent correlation between the sensor response and the mixtrue composition. Being highly sensitive and capable of analyzing both individual and mixed TICs, our gas sensor array has great potential for monitoring the safety and environmental effects of industrial chemical processes.

Langmuir adsorption isotherm for equation of sensor response to analyte gas concentration

The rescnce change ($\Delta R/R_0$) to analyte allows calibration curve which can be described by a kinetic adsorption model between analyte (A) and available adsorption site (θ) on the carbon nanotubes as follows

$$A + \theta \rightleftharpoons A\theta$$

Then, the equilibrium constant of analyte adsorption

$$K_{eq} = \frac{[A\theta]}{[A][\theta]}$$

The total adsorption site (θ_{total}) is the sum of the free site (θ) and adsorbed site ($A\theta$).

$$[\theta]_{total} = [\theta] + [A\theta]$$

Then, $[\theta]$total is expressed in terms of the analyte concentration $[A]$:

$$[\theta]_{total} = [\theta] + [A\theta] = [A\theta] + \frac{[A\theta]}{K_{eq}[A]} = [A\theta]\left(\frac{K_{eq}[A] + 1}{K_{eq}[A]}\right)$$

Assuming the resistance change of graphene is proportional to the $A\theta/\theta_{total}$ ratio,

$$\frac{\Delta R}{R_0} = a\frac{[A\theta]}{[\theta]_{total}} + b = a\frac{K_{eq}[A]}{1 + K_{eq}[A]} + b$$

where, a is proportionality factor, b is average background noise level, and n is Hill coefficient that represent cooperativity. Note that the value of b is 0 due to the normalized variation of sensor resistance.

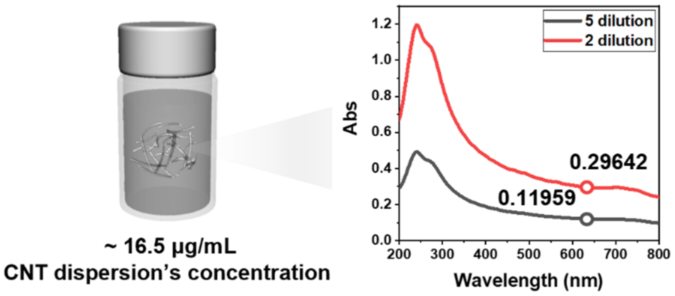

Figure S1 Coating material screening result compared to pristine SWCNTs sensor.

The absorption spectra of SWCNT dispersions were collected (Cary 5000, Agilent Technologies) to approximate to concentration of the SWCNT dispersion using the absorbance at 632 nm and an extinction coefficient of ε_{632} = 0.036 $(mg/L)^{-1}$ cm^{-1} [1]. The 2 and 5 times diluted SWCNT dispersion were measured at a wavelength range of 200 – 800 nm. The absorbance results of 0.29642 from 2x diluted and 0.11959 from 5x diluted solution at 632 nm suggest that the concentration of undiluted original SWCNT dispersion is 16.5 mg/L.

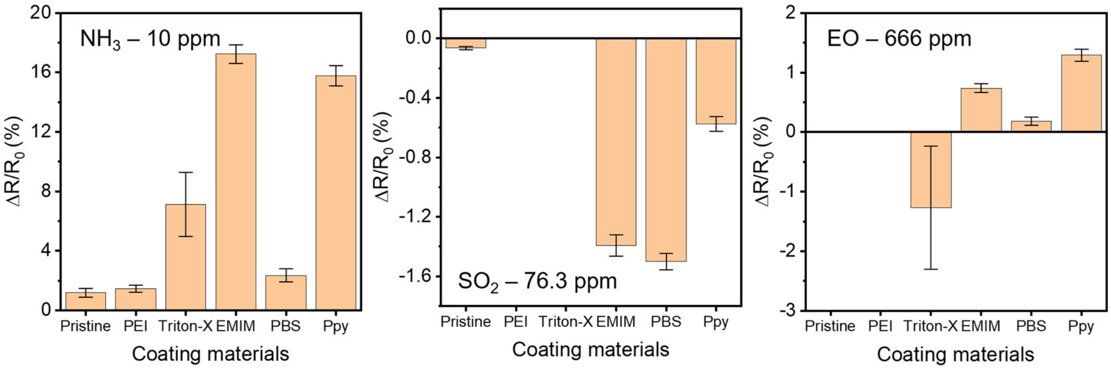

Figure S2 Coating material screening result compared to pristine SWCNTs sensor.

The sensor coated with EMIM exhibited the largest response to NH_3, while the sensor coated with PBS showed the largest sensitivity to SO_2. The sensor coated with Ppy demonstrated the highest response to EtO. Triton-X can be considered as a coating material for EtO sensing; however, it was excluded due to large deviation in response.

Baseline (Ω)	Sensor 1	Sensor 2	Sensor 3	Sensor 4	Sensor 5	Sensor 6	Sensor 7
**Initial R_0**	4671.642	2715.433	4753.795	1218.506	6974.185	2202.373	1233.561
**0.1 ppm**	4654.445	2715.466	4752.936	1220.193	6992.889	2206.845	1240.385
**0.2 ppm**	4626.263	2708.329	4739.847	1219.453	6974.774	2205.637	1240.184
**1 ppm**	4591.538	2695.338	4719.104	1217.182	6952.822	2202.316	1236.294
**2 ppm**	4557.914	2672.008	4667.966	1210.658	6883.282	2189.178	1222.972
**5 ppm**	4588.185	2698.985	4731.066	1220.24	6963.76	2209.335	1239.826
**AVERAGE**	4614.998	2700.926	4727.452	1217.705	6956.952	2202.614	1235.537
**STDEV.P**	39.62184	14.98842	29.20705	3.32273	35.11112	6.491878	6.135427

Figure S3 Baseline of EMIM-coated sensors after NH_3 exposures

The measurements of sensor response with increasing gas concentration were performed in one single device. Although the resistances of the baseline were fully recovered and the deviation is less than 1% (Table 1), small differences in baseline still exists because the baseline drift[2,3].

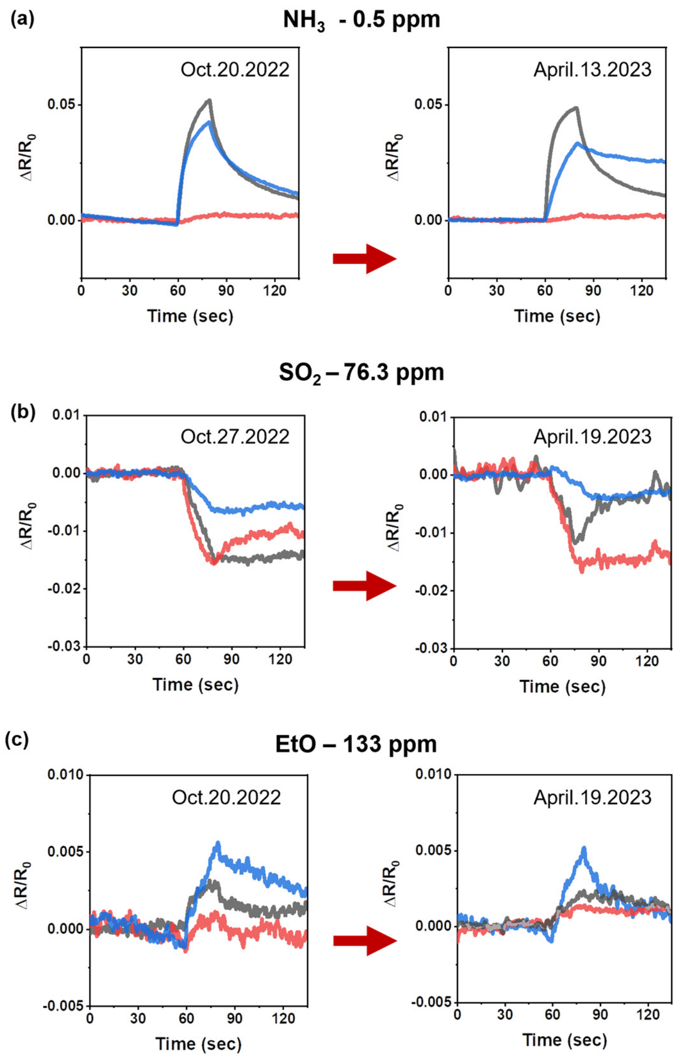

Figure S4. Stability of the sensor array. Sensor responses to NH_3, SO_2, and EtO were obtained after 6 months storage.

To prove the long-term stability of our SWCNTs chemiresistor array, the array used for sensing the gas in Figure 3 was re-measured after 6 months. The responses of the freshly prepared sensor array and after 6 months of storage in ambient atmospheric conditions was compared. When the array sensor was exposed to 0.5 ppm of NH_3, the response of Ppy-coated sensor slightly decreased whereas EMIM- and PBS-coated sensor showed almost similar responses after 6 months. When the sensor array exposed to 76.3 ppm of SO_2 after 6 months, the noise level of all sensors slightly increased. Furthermore, the irreversible response of EMIM-coated sensor turns to reversible while the reversible response of PBS sensor turns to irreversible. On the other hand, when the sensor array exposed to 133 ppm of EtO after 6 months, improved sensor noise was obtained while decreased response was obtained in EMIM-coated sensor. Although slight degradation in sensor performance was observed after 6 months storage at ambient environment, the detection of the target analytes at concentrations lower than the IDLH was available. However, considering the sensor signal change for quantitative analysis, further study is required for the long-term stability.

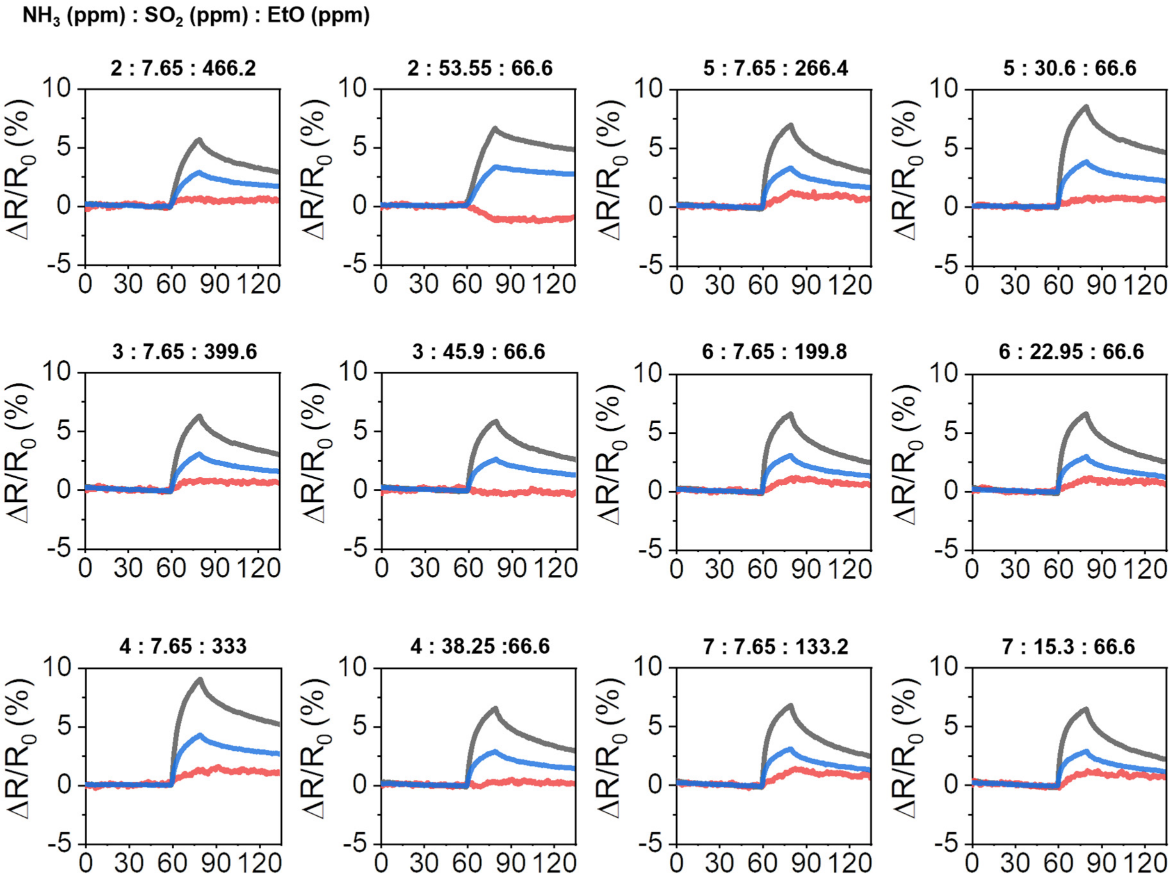

Figure S5 Sensor array responses toward three species gas mixtures.

1. Salem, D.P.; Gong, X.; Liu, A.T.; Koman, V.B.; Dong, J.; Strano, M.S. Ionic Strength-Mediated Phase Transitions of Surface-Adsorbed DNA on Single-Walled Carbon Nanotubes. *Journal of the American Chemical Society* **2017**, *139*, 16791-16802, doi:10.1021/jacs.7b09258.
2. Tang, R.; Shi, Y.; Hou, Z.; Wei, L. Carbon Nanotube-Based Chemiresistive Sensors. *Sensors* **2017**, *17*, doi:10.3390/s17040882.
3. Fennell Jr, J.F.; Liu, S.F.; Azzarelli, J.M.; Weis, J.G.; Rochat, S.; Mirica, K.A.; Ravnsbæk, J.B.; Swager, T.M. Nanowire Chemical/Biological Sensors: Status and a Roadmap for the Future. *Angewandte Chemie International Edition* **2016**, *55*, 1266-1281, doi:https://doi.org/10.1002/anie.201505308.
